# Editorial: Preterm birth and placental pathology

**DOI:** 10.3389/fendo.2023.1168185

**Published:** 2023-04-03

**Authors:** Sam Schoenmakers, Kjersti Aagaard, Liron Borenstein-Levin, Kondwani Kawaza, Lotte Elisabeth van der Meeren, Ben Willem Mol, Nathasha Raygaan Rhoda, Jill Shawe, Karel Allegaert

**Affiliations:** ^1^ Department of Obstetrics and Gynecology, Erasmus Medical Center (MC), University Medical Center, Rotterdam, Netherlands; ^2^ Department of Obstetrics and Gynecology, Division of Maternal Fetal Medicine, Baylor College of Medicine, Houston TX, United States; ^3^ Neonatal Intensive Care Unit, Ruth Rappaport Children’s Hospital, Haifa, Israel; ^4^ Department of Pediatrics and Child Health, Kamuzu University of Health Sciences, Blantyre, Malawi; ^5^ Department of Pathology, Erasmus Medical Center (MC), University Medical Center, Rotterdam, Netherlands; ^6^ Department of Pathology, Leiden Medical University Center (LUMC), Leiden, Netherlands; ^7^ Department of Obstetrics and Gynaecology, Monash University, Clayton, VIC, Australia; ^8^ Groote Schuur Hospital, Department of Paediatrics, University of Cape Town, Cape Town, South Africa; ^9^ Faculty of Health, University of Plymouth, Devon, United Kingdom; ^10^ Department of Development and Regeneration, KU Leuven, Leuven, Belgium; ^11^ South West Clinical School, Royal Cornwall Hospitals NHS Trust, Truro, United Kingdom; ^12^ Department of Pharmaceutical and Pharmacological Sciences, KU Leuven, Leuven, Belgium; ^13^ Department of Clinical Pharmacy, Erasmus Medical Center, Rotterdam, Netherlands

**Keywords:** preterm birth (PTB), inflammation, prevention, placental pathology, life course

Preterm birth is defined as any delivery occurring before 37 + 0 weeks of gestation, and is multifactorial in its origin. Unfortunately, it is still insufficiently understood and explored when considering etiologies (either spontaneous preterm labor in about two thirds of cases, or medically indicated preterm birth for pregnancy related or pre-existing maternal or fetal morbidities, about one third), and placental pathophysiology. While prematurity is the leading cause of mortality for children under the age of five worldwide, both the occurrence and burden are disproportionately experienced in low and middle income countries with rates varying from the lowest in well-resourced settings (8-11%) to the highest in low resourced settings (11-20%), like Malawi (1–3).

The World Health Organization estimates that about 15 million babies are born preterm annually, of whom about 1-3 million will die, and a relevant portion of survivors will have long-term morbidity during their life course. Preventive strategies, including preconception care, have the potential to optimize couples health before pregnancy with life course and intergenerational opportunities to prevent preterm birth and other neonatal morbidity.

When focusing on spontaneous preterm delivery, the underlying pathogenesis and molecular mediators are only partially understood. Associations with maternal risk factors (obstetric history, cervical surgery), intra-uterine or placenta related conditions (anatomical variation, infection, inflammation or ruptured membranes) or endocrine pathways have been described. However, associations have not been sufficiently strong to adequately predict and identify pregnant women at (high) risk for spontaneous preterm birth, so that currently interventions to prevent or avoid preterm birth remain ineffective. At present, the most robust evidence relates to tocolytic interventions aimed at delaying preterm birth and create a time window (48 h) to administer corticosteroids. This allows acceleration of fetal (lung) maturation and protection against cerebral damage with magnesium sulfate (4). Besides short term improved ‘lung’ maturation, there is also clear evidence that prenatal corticosteroids, when subsequently preterm birth is not prevented, reduces the risk of neonatal death and developmental delay in childhood (5).

The lack of understanding and insight into the pathophysiology of molecular mediators alternately rendering risk of preterm birth or protection to term birth results in real-life consequences for daily clinical practice. First, we primarily only have access to a very few and generally ineffective tocolytic interventions, instead of mechanism-driven therapies. Second, the limited capability to accurately or sensitively predict or identify women at risk for preterm birth leads to about 50% of pregnancies diagnosed as high risk pregnancies, deliver out of the expected time window, resulting in maternal, and more importantly fetal overexposure to antenatal corticosteroids. Prenatal overtreatment with steroids is a relevant concern, as there are animal experimental data (neurological, cardiovascular, renal, immune system) and human epidemiological data on an increase in neurobehavioral disorders during the life course in unnecessary former exposed cases that subsequently delivered at term equivalent age (6, 7).

While rupture of membranes often temporally accompanies spontaneous preterm birth, it is not universally true. However, preterm pre-labor rupture of membranes (PPROM) are a common issue associated with preterm delivery (8), with 25-40% of preterm births attributed to the permanent state of ruptured membranes. The roles of galectins in restoring the integrity of the damaged tissue and the healing of human membranes were reviewed, and Chen et al. reflected on this potential new approach on amnion regeneration. Another approach focuses on the potential role of immunomodulation to delay preterm contractions in the setting of preterm delivery. The impact of co-exposure to anti-inflammatory prostaglandins (15-Deoxy-Delta-12,14-prostaglandin J2) on inflammatory responses (interleukine-1β expression) in an *in vitro* cell model (myocytes, vaginal epithelial cells, primary amnion epithelial cells) has been quantified. The authors hereby concluded that the effects were cell-type dependent, with increased cell death for cells of vaginal or amniotic epithelial origin. Based on these results, Rasheed et al. concluded that this intervention is unlikely a useful approach. This re-illustrates the earlier described current setting of limited preventive measures, related to insufficient mechanistic understanding.

An additional area of clinical imprecision resulting in preterm birth surrounds placental pathophysiology. Jansen et al. reported on the risk of iatrogenic preterm birth for placenta previa or low-lying placenta, and how this might be mitigated by preventive interventions. Based on 34 studies retained in a systematic review and meta-analysis, the risks for preterm birth (<37, or <34 weeks of gestation), or very preterm birth (<32 weeks of gestation) were 46% versus 30%, and 17% versus 10% when placenta previa or low-lying placenta respectively were documented. While the risk has been quantified, data on beneficial value of cervical cerclage, pessary and intramuscular progesterone are lacking and inconsistent. Finally, and besides preventive strategies, we also need to better understand when preterm birth will, or will not, result in significant neonatal morbidity with impairments along the entire life course.

Related to this, Su et al. reported on an increased risk for respiratory complications in extremely preterm male infants. Using a propensity score matching approach, male extreme preterms as compared to gestational aged matched females had a significantly higher risk to develop bronchopulmonary dysplasia (any, or moderate to severe, 95 versus 84% and 39 versus 30% respectively). This at least suggest that sex-related endocrinological differences should be further explored, and that neonatal outcome should be reported according to fetal sex].

As reflected in the diversity of subjects discussed in this special topic, preterm birth is a research field still in search for better mechanistic understanding, to identify potential targets for prevention or treatment related interventions. This should improve the pregnancy and neonatal outcome. Therefore, it is also important to appreciate the sex, racial and social disparities in relation to risk of preterm birth and the accompanying morbidity and mortality.

To achieve this, a broad and multidisciplinary collaboration is necessary, and we hope that this Research Topic has contributed to this urgently needed approach.

## Author contributions

All authors listed have made a substantial, direct, and intellectual contribution to the work and approved it for publication.
